# Supplementary material for: DNA Fragmentation in Viable and Non-Viable Spermatozoa Discriminates Fertile and Subfertile Subjects with Similar Accuracy
Source: J Clin Med. 2020 May 4;9(5):1341. doi: 10.3390/jcm9051341 (PMC7290809; doi:10.3390/jcm9051341)
Supplement: Supplementary file 1 [file jcm-09-01341-s001.pdf]

Table S1. Binary logistic regression model with total sDF (Table 1A) or viable sDF (Table 1B) or non-viable sDF (Table 1C), and age, abstinence, sperm number, sperm progressive motility and sperm morphology as introduced variables. State variable was fertile vs. subfertile status.

**Table 1A**		**OR**	**95% CI**	***p* Value**
Total sDF		1.03	1.01-1.06	0.013
Sperm Number, millions/ejaculate	<39	1 (ref.)	-	-
	≥39	1.47	0.31-6.92	0.622
Progressive Motility, %	<32	1(ref.)	-	-
	≥32	4.32	1.10-17.00	0.036
Morphology, %	<4	1(ref.)	-	-
	≥4	2.79	1.18-6.58	0.019
Age, y	<38	1(ref.)	-	-
	≥38	0.48	0.23-0.99	0.047
Abstinence, d		1.30	1.05-1.63	0.018

**Table 1B**		**OR**	**95% CI**	***p* Value**
Viable sDF		1.06	1.03-1.10	<0.001
Sperm Number, millions/ejaculate	<39	1 (ref.)	-	-
	≥39	1.51	0.31-7.34	0.608
Progressive Motility, %	<32	1(ref.)	-	-
	≥32	4.96	1.23-20.01	0.024
Morphology, %	<4	1(ref.)	-	
	≥4	3.32	1.36-8.06	0.008
Age, y	<38	1(ref.)	-	-
	≥38	0.49	0.23-1.05	0.065
Abstinence, d		1.33	1.06-1.66	0.014

**Table 1C**		**OR**	**95% CI**	***p* Value**
Non-viable sDF		1.07	1.04-1.10	<0.001
Sperm Number, millions/ejaculate	<39	1 (ref.)	-	-
	≥39	1.35	0.25-7.23	0.722
Progressive Motility, %	<32	1(ref.)	-	-
	≥32	8.14	1.62-40.89	0.011
Morphology, %	<4	1(ref.)	-	-
	≥4	3.21	1.27-8.10	0.013
Age, y	<38	1(ref.)	-	-
	≥38	0.51	0.24-1.10	0.087
Abstinence, d		1.21	0.96-1.53	0.112
